# AMR and Covid-19 on the Frontline: A Call to Rethink War, WASH, and Public Health

**DOI:** 10.5334/aogh.3140

**Published:** 2021-02-24

**Authors:** Mark Zeitoun, Ghassan Abu Sittah, Reem Shomar, Nassim el Ach

**Affiliations:** 1 Water Security Research Centre, University of East Anglia, Norwich NR4 7TJ, UK; 2 Conflict Medicine Programme, American University of Beirut, LB; 3 Islamic University of Gaza, PS

## Abstract

This Viewpoint calls for a greater understanding of the role that water plays in the transmission of anti-microbial resistance and covid-19 in protracted urban armed conflict, in order to develop a ‘pathogen-safe’ practice. It argues that dealing with the twin threats is difficult enough in the best of circumstances, and is so little understood in war zones that surgeons and water engineers now question if their practice does more harm than good. Experience suggests that the known transmission routes are complicated by a great number of factors, including the entry of heavy metals through bullets in patients’ wounds, hospital over-crowding, mutation in treated water or wastewater, and other threats which endure long after the bombing has stopped. The skeleton research agenda proposes greater sewage surveillance, testing of phages and monitoring of treatment designed to dispel or substantiate these assertions.

## The challenge

If covid-19 is indeed accelerating the perils of anti-microbial resistance (AMR) in the US and UK, [1] it has surely sent them beyond warp-speed in the war zones of the Middle East. The challenges that we, water engineers and surgeons, face on the frontline were daunting enough before the dawn of covid-19. Now, we suspect that the drugs which we prescribe and the water we treat may actually be harming rather than helping.

The rampant virus and the mutating bacteria must force a change to the traditional public health response of agencies that are active in humanitarian contexts. This obliges no less than a re-conception of practice, and can come only after development of the knowledge and policy base.

## Runaway mutations and antibiotic use

The ‘antibiotics apocalypse’ has already claimed hundreds of thousands especially in low and middle-income countries [2]. Responses which have been developed to deal with covid-19 can learn a lot from our stumbling in the dark with AMR from Aleppo to Tripoli, through Gaza and Aden. Here, resistant bacteria can come in on a sniper’s bullet [3], mutate via surgical instruments that are disinfected in contaminated water [4], and be carried back to the community through the hospital’s wastewater pipes [5].

We lose pan-drug resistant patients every day when the fighting is most intense, and every week between the bouts of violence, as these continue to be shaped by ongoing tensions and economic sanctions. Consider the eight-year-old girl from Aleppo who came in February 2020 with bilateral above-knee amputations and burns, and who died of infections despite the tetracycline and colistin we gave her. Or the hundreds of teenagers shot in the knees who overwhelmed hospitals in Gaza in March 2018 [6]. Many of those wounds have been re-infected three times since then, despite repeated doses of antibiotics.

Covid-19 amplifies the manslaughter when the family that cannot be kept out of the wards returns to care for their injured, and even *more* antibiotics are dispensed to covid-19 patients [7].

## Where’s the advice?

**Figure 1:**
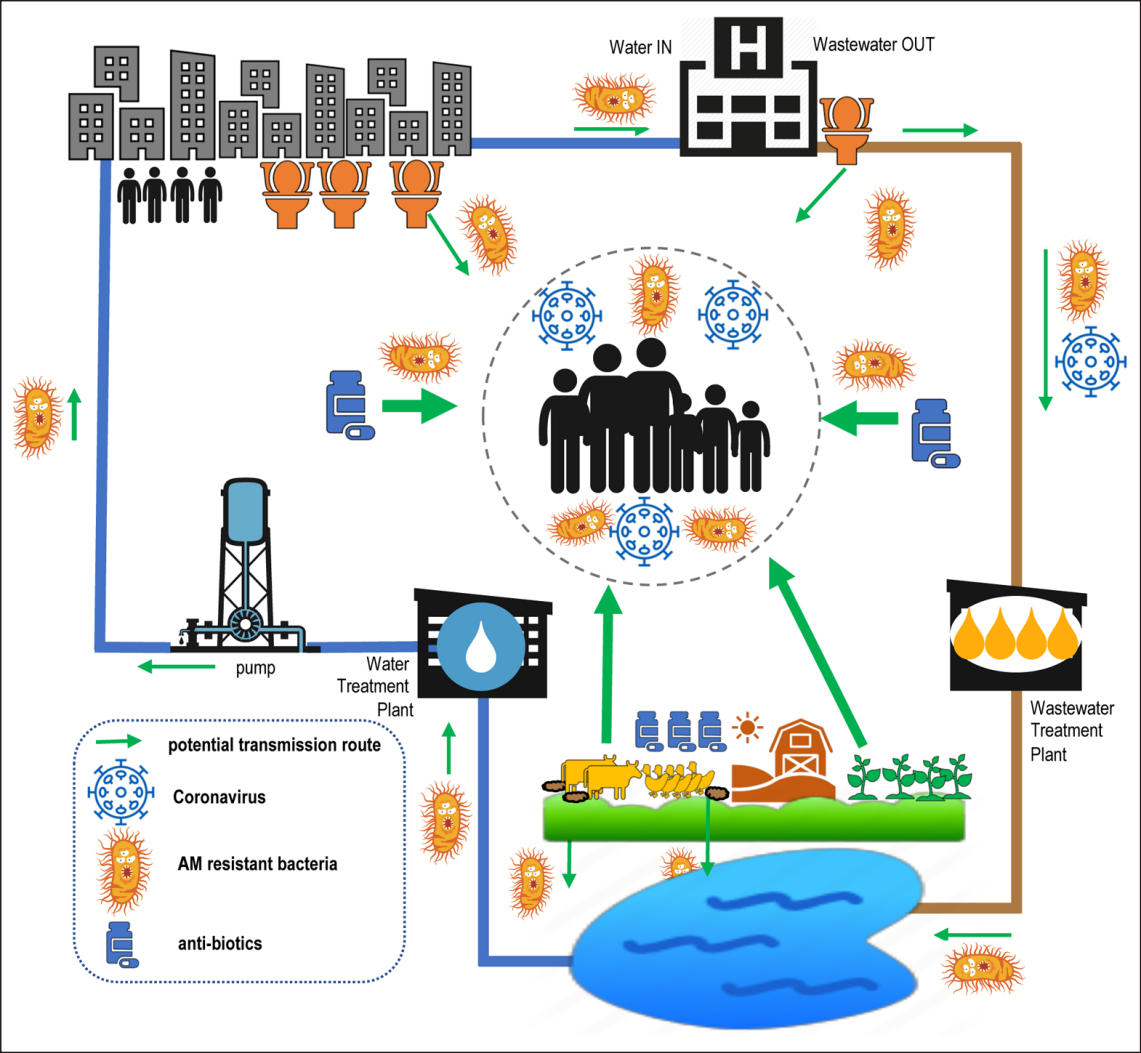
Suggested transmission routes of AMR and covid-19, in a typical urban (non-conflict) setting. Width of arrows indicates suggested relative magnitude of importance.

**Figure 2:**
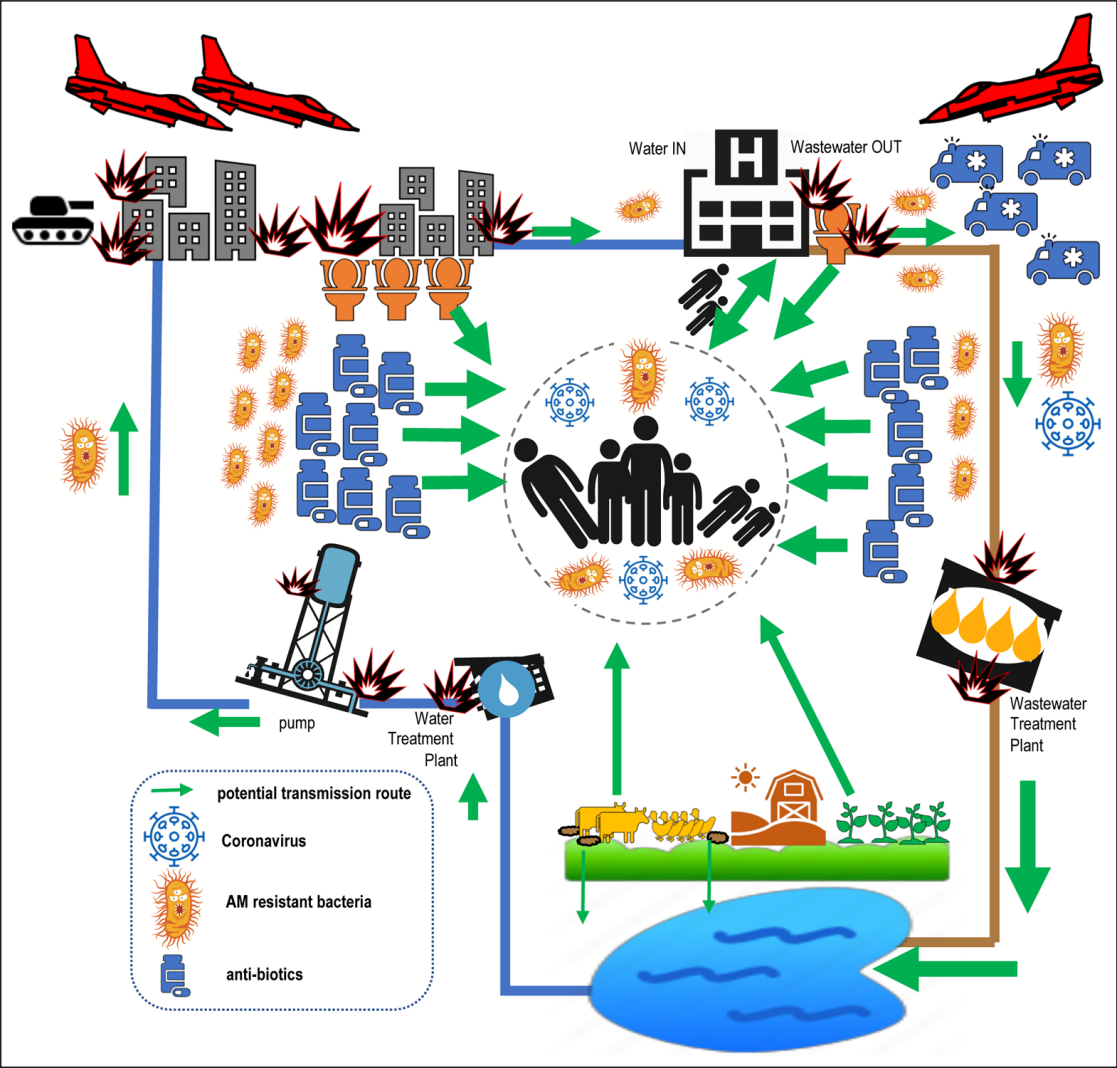
Hypothesized transmission routes of AMR and covid-19, in a typical setting of protracted urban armed conflict. (from *WASH and AMR in protracted conflict* group at AUB). Width of arrows indicates suggested relative magnitude of importance.

We know that standard drinking water and wastewater treatment may get the virus [8], but not the bug [9]. Of the bigger picture, we do not know a whole lot more. The World Health Organization (WHO) Global Strategy for Containment of AMR is strong on Health Care Facilities (HCF), for instance, but makes almost no mention of water sanitation and hygiene (WASH). Status updates on WASH in HCF miss out on AMR [10], while reports on WASH and AMR omit hospitals [11]. The breadth of the interdisciplinary One Health WASH in Health Care Facilities and similar initiatives stand out amidst this field, and have suggested numerous transmission route diagrams, as in ***Figure 1*** [12,13,14,15,16]. Gaining a better understanding of these pathways is of crucial importance in most ‘normal’ contexts, even refugee camps [17]. But the known pathways are less relevant where the backdrop sees heavy metals on shrapnel rip into flesh, economic sanctions pervert access to pharmaceutical markets, and pathogens define an entire define a ‘biosphere’ of endless armed conflict – as in ***Figure 2*** [18,19,20,21,22].

In the disastrous ‘ecology of war’ which characterizes protracted conflicts, the impact of sanctions goes well beyond the blast zone, and endures long after the dust settles [23,24]. We have to guess if the persistent infections stem from the water used to disinfect the scalpel, or the soap used to wash our hands [25,26]. After all, they may just as well be a result of the fact that we run out of drugs [27], or have to prescribe drugs which are counterfeit [28,29], or which have been kindly donated but do not meet WHO guidelines (like the near-expiry Carbapenems dumped in Gaza in 2018) [30]. In these contexts, patients are exposed in equal measure to viruses, superbugs, cephalosporins [31], and missiles exploding in the rooms they have come for consultation. Hospitals become petri-dishes; carers become carriers. ‘Clean’ water infects, soap kills.

## Research towards ‘pathogen-safe’ practice

Blind of insight, we double-down on scrubbing-up, over-chlorinate the water, treat the wastewater, rebuild antibiotic stewardship programs and blend them with covid-safe practice, scream at families to social-distance, track-and-trace, hope and pray. To little apparent avail. The patients keep coming and our options keep narrowing.

Of course, better disinfection and more beds will help, but only a knowledge base will help us get ahead in the game. We first need to establish which of the transmission routes proposed in ***Figure 2*** are the most relevant to covid-19 and AMR. We must determine if the direct link between the conduct of hostilities and diarrhea multiplies or (somehow) reduces AMR [32], for example – meaning more ‘sewage surveillance’ of health care facilities should take priority [33,34].

We must then determine the best ways to prevent or interrupt the pathways. What is the scope for bacteriophages [35], for example, especially if they can be cultivated in the kind of labs which still function in war zones? We will need better monitoring of water treatment, too, to determine if reverse-osmosis is as pure and safe as we would like to think, or dangerous, as the biofilm accumulates on the membrane become a grand reservoir for mutation [36].

The answers can lead to policy that guides ‘pathogen-safe’ practice in war zones, and improves the quality of life of hundreds of millions [37]. Until then, we accept our work might be doing some serious harm – and call researchers of all stripes from the sidelines to the front line.
